# Biocontrol activity and action mechanism of *Bacillus velezensis* strain SDTB038 against Fusarium crown and root rot of tomato

**DOI:** 10.3389/fmicb.2022.994716

**Published:** 2022-09-02

**Authors:** Qiqi Chen, Yue Qiu, Yazhen Yuan, Kaiyun Wang, Hongyan Wang

**Affiliations:** Department of Plant Protection, Shandong Agricultural University, Tai’an, Shandong, China

**Keywords:** *Bacillus velezensis*, Fusarium crown and root rot, tomato, control mechanism, genome analysis

## Abstract

Fusarium crown and root rot of tomato is a soilborne diseases that has brought serious harm and economic losses to tomato production in facilities in recent years. The disease has been reported in more than 30 countries worldwide, but there are few reports on its biological control. A *Bacillus velezensis* strain SDTB038 with biocontrol effects was isolated and identified in a previous study and is considered one of the most important PGPRs. Seven secondary metabolite biosynthesis gene clusters were found in strain SDTB038 by whole genome sequencing, explaining its biocontrol effects. Results indicated that different concentrations of SDTB038 fermentation broth inhibited the mycelial growth of Fusarium crown and root rot of tomato. Strain SDTB038 could generate indole acetic acid and promote healthy growth of tomatoes, while the effect of 10^8^ CFU/ml SDTB038 concentration on promoting tomato growth was the most obvious. *B. velezensis* SDTB038 significantly reduced the accumulation of ROS in tomato plants, induced the up-regulation of antifreeze genes, and promoted the rapid recovery of tomato plants at low temperatures in a pot experiment. At the same time, SDTB038 had good control effect on Fusarium crown and root rot of tomato, and 10^8^ CFU/ml SDTB038 fermentation broth had the best control effect, which was 42.98%. In summary, the strain *B. velezensis* SDTB038 may be a promising bacterial agent for biological control of Fusarium crown and root rot of tomato, and an important source of potential antimicrobial compounds.

## Introduction

Tomato (*Solanum lycopersicum*) is a common gardening crop. In recent years, the tomato planting area has increased, along with yields (Cheng et al., 2021). However, the frequent occurrence of tomato diseases affects yield and quality, and soilborne diseases are particularly difficult to control because they are persistent and widespread (Borisade et al., 2017). Fusarium crown and root rot of tomato caused by *Fusarium oxysporum* f. sp. *radicis-lycopersici* is a severe soilborne disease (Mousa et al., 2021). *F. oxysporum* is the pathogen causing this disease and tomato Fusarium wilt, but the pathogens show different specializations. Tomato Fusarium wilt is caused by *F. oxysporum* f. sp. *lycopersici*. Although both of these pathogens can cause plant wilting, there are great differences in the associated symptoms, infection period, conditions and host infection (Bharti et al., 2017). Fusarium crown and root rot of tomato easily occurs under low temperature and high humidity, and the pathogen is mainly transmitted through soil, diseased plants, diseased seeds or compost (Rekah et al., 2000). The disease has seriously restricted the development of facility production of tomato (Deng et al., 2022). Although it is a very damaging pathogen to tomatoes, the management strategies for controlling the pathogen are limited (Cao et al., 2018), and there is an urgent need for a better way to control this disease to ensure production safety, reduce losses and increase outputs (Chu et al., 2021).

Fusarium crown and root rot of tomato are mainly controlled by biological control, which had proven the best method and received increasing attention from the agricultural industry in recent years. Plant growth-promoting rhizobacteria (PGPR) are bacteria that are good for crops and can stabilize the soil, promote plant growth and inhibit or reduce plant damage caused by disease (Joly et al., 2021). PGPR are considered promising biocontrol agents and utilize the plant rhizosphere and other areas as attachment points; these organisms have a control effect on pathogens, which could reduce the incidence of diseases (Riaz et al., 2021). The main biological control mechanisms include competition and plant growth promotion (Zhang et al., 2021). *Bacillus* had become one of the most studied PGPRs in recent years due to its wide variety of sources, high natural survival rate, inhibition of a variety of plant pathogens and environmental friendliness (Aly et al., 2022). Moreover, because it could produce secondary metabolites with antimicrobial activity, it promoted plant growth as a biological agent to control diseases over large areas (Ma et al., 2022).

*Bacillus velezensis* is an important PGPR that can effectively control soilborne diseases and ensure the normal growth of plants. Dhouib et al. (2019) showed that *B. velezensis* is a good biocontrol strain and could effectively control tomato verticillium wilt. Vignesh et al. (2022) showed that *B. velezensis* NKMV-3 isolated from the rhizosphere had a good biocontrol effect against *Alternaria solani*. Through genome-wide prediction, we found that the strain had seven gene clusters for secondary metabolite biosynthesis. Secondary metabolites are closely related to antimicrobial activity. *B. velezensis* had antimicrobial activity and could produce indoleacetic acid, which has good biocontrol potential (Yan Y. et al., 2021). Therefore, *B. velezensis* has good biocontrol potential against many tomato diseases, but there are few reports of its effects on Fusarium crown and root rot of tomato.

Low temperature is one of the most common abiotic stresses in plants around the world. Inhibition of plant growth and weakening of plant light cooperation have become one of the main factors restricting agricultural production (Wang et al., 2020). Under low temperature conditions, plants respond to external conditions through a series of biochemical reactions, such as leaf wilting, weakened photosynthesis, large accumulation of reactive oxygen species (ROS) in plant cells and reduced enzyme activity (Han et al., 2020). However, in order to reduce the oxidative damage caused by ROS accumulation in plant cells [mainly superoxide anion (O^2−^) and hydrogen peroxide (H_2_O_2_)], biological or non-biological factors could be used to regulate the content of related enzymes in plants or induced the expression of antifreeze genes (Liu et al., 2016). Improving the freezing resistance of crops, eliminating the accumulation of ROS in them and protecting the healthy growth of crops are a key goal of agricultural production.

In view of the above facts, this study aimed to determine the inhibitory activity of SDTB038 fermentation broth with different concentrations against *F. oxysporum* f. sp. *radicis-lycopersici*, the promoting effect of SDTB038 on tomato seed germination and plant growth, the colonization dynamics of SDTB038 in soil and plants, and the field control effect of SDTB038 fermentation broth on Fusarium crown and root rot of tomato. The SDTB038 strain reduced the accumulation of low-temperature-induced ROS in tomato leaves, promoted the rapid recovery of plants at low temperature, and increased the expression of stress resistance genes. The whole genome of SDTB038 was sequenced, and its biosynthetic gene clusters were predicted. The reasons for the biocontrol potential of *B. velezensis* SDTB038 were explained at the genomic level, and the biocontrol mechanisms were preliminarily explored, providing a theoretical reference for future studies and further applications.

## Materials and methods

### Whole-genome sequencing analysis of SDTB038

DNA extraction and quality detection of *B*. *velezensis* SDTB038. A strain of *B. velezensis* SDTB038 with biocontrol potential was isolated in our laboratory and preserved in the China General Microbiological Culture Collection Center (CGMCC) under CGMCC no. 19215 (Yan H. et al., 2021). *B*. *velezensis* SDTB038 was cultured to logarithmic phase. Genomic DNA was extracted using a bacterial DNA kit (Tiangen). The concentration of genomic DNA extracted from SDTB038 was determined by an Epoch2 enzyme-labeled instrument (BioTek, United States). The qualified samples are transferred to Beijing concentration were sent to Novogene Co., Ltd. for detection.

Gene prediction and annotation. GeneMarkS software (verison4.17)^1^ was used for coding genes expection, statistical genes expection results. The predicted genes were compared with GO, KEGG, COG and other functional databases by BLAST. The annotations of identity and coverage ≥40%, e-value ≤1e^−5^ in the sequence BLAST results were selected, and the detailed information obtained by merging all gene annotations was sorted out.

### Isolation and identification of pathogens

Using the conventional tissue separation method (Mousa et al., 2021), the isolated tissue was cultured at 25°C for 2–3 days, and the marginal mycelia were selected and transferred to a new PDA plate for preliminary purification. A single spore was selected for purification and cultivation, and the colony and spore morphology were observed (Khaskheli et al., 2020). The isolated and purified strains were cultured on PDA plates and exhibited round colonies, white, villous mycelium, and the colony color changed to light pink later. Under the microscope, the two heads of macroconidia were pointed, slightly curved, sickle-shaped, with septa; microconidia were elliptical, septate. A spore suspension was prepared according to Nekoval et al. (2022). After root injury, each tomato seedling was irrigated with 10 ml spore suspension, and the symptoms were observed after the disease.

According to the Phanta Max Super-Fidelity DNA Polymerase (Vazyme Biotech Co., Ltd., Nanjing, China) kit requirements, *ITS* gene sequences were amplified and sequenced by Sangon Biotech (Shanghai) Co., Ltd. The sequencing results were analyzed by the Blast program in the NCBI database, and the strains were preliminarily classified and identified (Obunukwu et al., 2018). MEGA 7.0 software was used to construct a phylogenetic tree, and the *Sprl* gene fragments of the tomato Fusarium wilt pathogen and other isolates were amplified with Sprlf and Sprlr primers for verification (Table 1). *Sprl* gene fragments of the tomato wilt pathogen (FOL) and isolated strain (FO-1) were amplified by PCR and detected by 2% agarose gel electrophoresis.

**Table 1:** Primers and sequences used for PCR.

Primer name	Sequences (5′-3′)
Sprlf	GATGGTGGAACGGTATGACC
Sprlr	CCATCACACAAGAACACAGGA

### Antimicrobial effects of SDTB038

A single colony of SDTB038 was cultured in sterilized LB. The seed solution was prepared at 25 ± 1°C at 180 r/min for 12 h (Cejas et al., 2012). The seed solution was transferred to a new sterilized conical flask with a 1% inoculation amount and cultured at constant temperature for 14 h. The fermentation broth was adjusted to 10^10^ CFU/ml with sterilized liquid LB and then diluted to 10^9^, 10^8^, 10^7^, 10^6^, 10^5^ and 10^4^ CFU/ml. A sterile punch (*d* = 5 mm) was used to remove a plug from the edge of the pathogen colony and transfer it to the center of a PDA plate. One microliter of fermentation broth was extracted with a pipette and placed on both sides of the mycelial plug (Villarino et al., 2021). With LB as the control, the mycelia grew to 3/4 of the plate at 25°C, and the test effects were observed.

### Determination of the growth temperature of SDTB038

The preparation of the biocontrol bacterium SDTB038 seed liquid was as described above. A 10 ml sterile centrifuge tube was added to 3.92 ml sterile liquid LB and 80 μl seed liquid in each tube, and the same amount of liquid LB was added as a control. After sealing the membrane, the tube was placed in a constant temperature oscillator at different temperatures (5, 10, 15, 20, 25, 30, 35, 40, 45, 50°C) and 180 rpm. After 12, 24 and 48 h of culture, the OD value at 600 nm wavelength was measured by microplate reader and the ΔOD600 was calculated.

### Determination of the production capacity of growth-promoting substances of SDTB038

Detection of IAA secretion activity: SDTB038 was cultured in 20 ml liquid LB containing 1% L-tryptophan (Buy from Beijing Solarbio Science & Technology Co., Ltd.) for 2 days (180 rpm/min, 25°C) without biocontrol bacteria as a control. Two milliliters of supernatant, was added to 4 ml of Salkowski reagent and two drops of phosphoric acid. The mixture became red, proving that SDTB038 could secrete IAA (Jangir et al., 2018).

Phosphate dissolution activity test: sterile filter paper (5 mm) was placed in the center of NBRIP plate, and 2 μl bacterial solution was added dropwise on the filter paper. Cultured at 25°C for 7 days, if transparent halos appear around colonies, SDTB038 can dissolve phosphate.

### Water culture experiment with tomato seedlings

Tomato seeds were germinated in a seedling tray containing sterile matrix soil, cultured in a light incubator at 25°C ± 1°C, and managed with normal watering and fertilization. When the seedlings had formed two leaves and one center, cultivated seedlings were transplanted into the greenhouse of Shandong Agricultural University for hydroponics cultures (Gao et al., 2018). A white plastic square box was used in the hydroponic container, and the container was filled with sterilized Hoagland nutrient solution. The tomato seedlings were fixed with a foam floating plate, and three seedlings were transplanted into each box (the substrate attached to the rhizosphere was carefully removed when the seedlings were transplanted into sterile water). The following seven treatments were set up (30 ml per box per treatment): A: CK (water control) B: 0.01 mg/l S-inducible hormone treatment (spray); C: LB treatment; D: 10^7^ CFU/ml SDTB038; E: 10^8^ CFU/ml SDTB038; F: 10^9^ CFU/ml SDTB038; G: 10^10^ CFU/ml SDTB038. After transplanting, the seedlings were treated once every 7 days for the first time continuously treated 3 times, and the nutrient solution was replaced once every 7 days. Three repetitions were performed per treatment (Souri and Tohidloo, 2019). The height, stem thickness, root length and effective leaf number of tomato seedlings were measured and recorded at 7, 14, 21 and 40 d after the first test group, and the fresh weight and dry weight of plants and roots were weighed at 40 d. After 30 min of treatment at 105°C in an oven to devitalize the green tissues, samples were dried to constant weight at 80°C to determine the dry weight (Singh et al., 2016).

### Real-time PCR detection and histochemical detection of H_2_O_2_ and O^2−^

Two treatments were established in the experiment: 1: LB control, 15 ml liquid LB medium was applied to each plant; 2: Application of SDTB038 fermentation broth, 15 ml 10^8^ CFU/ml SDTB038 fermentation broth was applied to each plant. Colonization at room temperature for 48 h, was allowed under a 4°C low temperature treatment. There were three replicates per treatment, and three plants per replicate. After treatment, the leaves of the control and treatment groups were taken at 0, 3, 6, 9, 12 h, 1 h rewarming and 3 h rewarming and stored at −80°C for total RNA extraction. An RNA extraction kit and reverse transcriptase kit were used for total RNA extraction from tomato leaves and reverse transcription. The qRT-PCR used SYBR Green Mix reagent kits according to the manufacturer’s instructions (The kits are purchased from Vazyme Biotech Co., Ltd., Nanjing, China). QRT-PCR primers were designed by Shanghai Sangon Biological Technology and Services Co., Ltd. (Shanghai, China; Table 2).

**Table 2:** Primers used for qRT-PCR.

Gene name	Forward primers (5′-3′)	Reverse primers (5′-3′)
*SIMPK3*	GCAACTCCCACAACATCC	TCTGCTCTTCTCCTATCCCT
*SIHSP17.7*	CACCGAAGGAGGAAGGAAAGTGG	TTTGCGTTCTCTGGAAGTC
*SLMYB7*	CAGATGCCCAAATTCGCAGG	CTGCTGCAGGGTGAACAAAC
*SICPK8*	CTCTAGAATGAGTAGCTCAACGTCAACGC	GGGTACCTTAAGACCCTTTTTCTTCAGAG
*SIHY5*	GGCTCTAGAATGCAAGAGCAAGCGACG	CCATGGGCTTCCTCCCTTCCTGTGCACC
*SOD*	GGCACCATCCTCTTCACTC	GCACCATGCTCCTTACCAG
*CAT*	GATGAGCACACTTTGGAGCA	TGCCCTTCTATTGTGGTTCC

The *in situ* formation of H_2_O_2_ and O^2 -^ in the leaf was detected according to the method of Zhang et al. (2019). Diaminobenzidine (DAB) and nitroblue tetrazolium (NBT) were purchased from Beijing Solebo Technology Co., Ltd. After staining, the cells were allowed to stand for 12 h to decolorize. Each treatment was repeated three times (Han et al., 2020). At the same time, a hydrogen peroxide kit and superoxide ion kit were used to measure the H_2_O_2_ and O^2−^ contents. Kits were purchased from Suzhou Geruisi Biotechnology Co., Ltd.

Determination of the H_2_O_2_ content:approximately 0.1 g tomato leaves were weighed, 1 ml acetone was added, the mixture was homogenized in an ice bath, transferred to an EP tube, brought to constant volume of 1 ml with acetone, and centrifuged at 12000 rpm and 4°C for 10 min; the supernatant was retained and placed on ice for subsequent measurement.

Determination of the O^2−^ content:approximately 0.1 g tomato leaves were weighed, 1 ml of extract was added, the mixture was homogenized in an ice bath, and then centrifuged at 12000 rpm and 4°C for 10 min; the supernatant was retained as crude body fluid and placed on ice for subsequent measurement.

### SDTB038 colonization test

Determination of natural resistance of the biocontrol strain SDTB038. Preparation of drug-containing plates containing kanamycin, rifampicin and ampicillin at final concentrations (All purchased from Beijing Solarbio Science & Technology Co., Ltd.) of 20, 50 and 100 mg/l, and the plates without drug additions were used as the control. The SDTB038 fermentation broth was diluted in 100 μl, absorbed by a pipette and evenly coated on the plate. SDTB038 was cultured at 25°C for 12 h to examine the growth of SDTB038.

Determination of rifampicin resistance of soil microbial populations. Tow grams of soil was dissolved in water and diluted 100 times. Then, 100 μl of the soil suspension before and after dilution was evenly coated on the LB plate containing 100 mg/l rifampicin, and the LB plate without rifampicin was used as the control. The growth of the soil microbial population on LB plates containing rifampicin was observed at 25°C for 10–12 h (Schreiter et al., 2018).

Induction of rifampicin-resistant strains. The concentrations of the rifampicin resistance induction series were set as follows: 0.1, 0.5, 1.0, 2.0, 4.0, 8.0, 16.0, 25.0, 50.0, 100.0 and 200.0 mg/l. Single colonies were picked and cultured in liquid LB containing 0.1 mg/l rifampicin for 12 h, streaked on LB plates containing the same concentration of rifampicin and cultured at 25°C. Single colonies with the same shape as the original colonies were selected and transferred to liquid LB plates containing lower concentrations of rifampicin to continue shaking culture until the induced strains could grow normally on LB plates containing 200 mg/l rifampicin (Stoll et al., 2021; Huang et al., 2022).

Determination of the genetic stability of rifampicin resistance of the SDTB038 strain. After rifampin resistance induction, the resistant marker strain was transferred to an ordinary LB plate for 3 generations by the streaking method, and then transferred to the LB plate containing 200 mg/l rifampin to detect its genetic stability (Rathore et al., 2016).

Morphological observation, physiological and biochemical characterization and molecular biological identification of the rifampicin-labeled strain. Morphological characteristics: Rifampicin-resistant labeled strains and wild-type strains were lined up on LB plates at the same time, and single colonies were cultured on LB plates for later observation and recording (Wang et al., 2022).

Molecular biological identification: Total DNA of labeled and wild-type strains was extracted by a bacterial DNA kit (Vazyme Biotech Co., Ltd., Nanjing, China). The primers were sequenced by Sangon Biotechnology (Shanghai) Co., Ltd. The 16S rRNA gene and *gyrB* gene were amplified by using universal primer sets (27F and 1492R; UP1f and UP2r; Table 3), and DNMAN was used to compare the gene sequences of the two strains to determine the relationship between rifampicin-resistant marker strains and wild strains (Soergel et al., 2012).

**Table 3:** Primers and sequences used for PCR.

Primer name	Sequences (5′-3′)
27F	AGAGTTTGATCMTGGCTCAG
1492R	TACGGYTACCTTGTTACGACTT
UP1f	GAAGTCATCATGACCGTTCTGCAYGCNGGNGGNAARTTYGA
UP2r	AGCAGGGTACGGATGTGCGAGCCRTCNACRTCNGCRTCNGTCAT

SDTB038 colonization and SEM observation. Thirty ml of rifampin-resistant marker strain fermentation broth was inoculated in each pot by the root irrigation method. With the plants watered with 30 ml sterile liquid LB as the control, the rhizosphere soil, root tissue, stem base and leaves of tomato plants were taken 1, 2, 3, 4, 5, 6, 7, 14, 21, 28 and 60 d after root irrigation. The labeled strains were recovered from a plate containing 100 mg/l rifampicin, and the colonization of the labeled strains at different times and in different plant parts was analyzed (Chu et al., 2021). Scanning electron microscopy was performed according to a previous method (Guo et al., 2017). The colonization of strain SDTB038 in roots and stems of tomato seedlings at 7 d and 60 d was observed by scanning electron microscopy.

### Field test

The field experiment site was located in Fangcun Beibudong Village, Daiyue District, Taian City, Shandong Province, China. Tomato is the main greenhouse vegetable in Fangcun Village, and it is planted in both spring and autumn. The greenhouse selected in this experiment had been in tomato cultivation for more than 20 years. The test site had good soil and fertility conditions, tillage measures in place, and easy-access to irrigation. The tomato seedlings were planted on August 31, 2020. The plant spacing in the shed was approximately 55 cm, and the row spacing was approximately 1.1 m. Two thousand tomato seedlings were planted per mu.

The experiment used a randomized block distribution, with a total of 20 plots. The plot area was 21 m^2^, and each treatment set had 5 replicates, with the treatment interval of a plot for the isolation zone. The following four treatments were set: 1: blank control, no application of biocontrol bacteria and pesticides; 2: LB control, 10 ml liquid LB medium was applied to each strain; 3: Microbial fertilizer treatment (content of *Bacillus subtilis* in microbial fertilizer ≥20 billion CFU/g, 0.5 g per plant); 4: Chemical treatment, 75 g/ha 10% difenoconazole water dispersible granules (Buy from Shandong Biao Biotechnology Co., Ltd.); 5: Application of SDTB038 fermentation broth, each strain was treated with 10 ml 10^8^ CFU/ml SDTB038 fermentation broth. The base of the stem was sprayed with a 3 WBD-20 electric sprayer in the difenoconazole treatment, and 450 l/ha water was used for each treatment. The fermentation broth of biocontrol bacteria was used to irrigate the roots (Luo et al., 2019). A total of 3 applications of biocontrol bacteria were made, at transplanting (August 31); before cooling (November 21); and 7 days after the second treatment. After the obvious onset of disease in the control treatment, the disease incidence under each treatment was checked and recorded, and the disease index (DI) and disease control effect (DCE) were calculated.

Disease classification standards are as follows: level 0: plant health, no disease; level 1: roots brown, not soft rot, not constricted, leaves healthy, root no obvious lesion; level 2: the root became brown with obvious constriction, the tip or leaf became yellow, and the root became brown; level 3: the roots became brown and rotten, the leaves became yellow, and the roots became brown or even black; level 4: root and root rot, whole seedling necrosis (Ma and Wehner, 2015).

Disease index (%) = (∑ number of plants of all grades × representative value of disease classification)/highest disease level × all number of observed plants × 100.

Control effect (%) = ∑ (blank group disease index−treatment group disease index)/blank group disease index × 100.

### Data analysis

The data obtained in the antibacterial effect test of lipopeptide crude extract were used to calculate the median lethal concentration (EC_50_) by ANOVA in Excel and SPSS 18.0 software. Indoor germination, greenhouse growth and field test data were statistically analyzed by Duncan New Complex Range (DMRT), and expressed as mean ± standard deviation (SD).

## Results

### Genome-wide sequencing analysis of strain SDTB038

**Figure 1:**
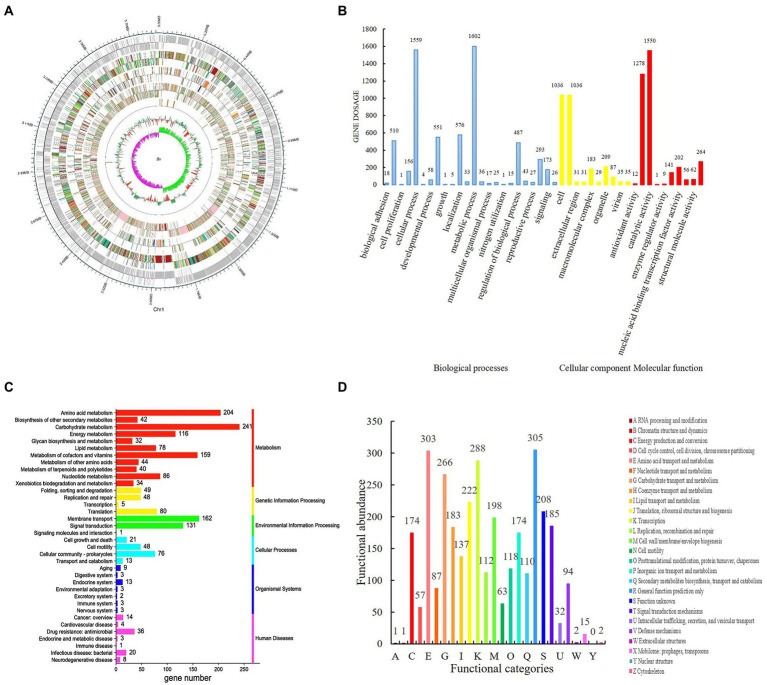
Genome-wide analysis of *Bacillus velezensis* SDTB038. **(A)** genome circular map of SDTB038 strain; **(B)** Gene Ontology (GO) sub-classification of SDTB038. Blue: biological process; Yellow: cellular components; Red: molecular functions; **(C)** Statistical chart of KEGG secondary function classification. Red: metabolism; Yellow: genetic information processing; Green: environmental information processing; Light blue: cellular process; Dark blue: organismal system; Light red: human disease. **(D)** Functional classification map of strain SDTB038 in COG.

Genome-wide data for *B. velezensis* SDTB038. To understand the strain SDTB038 more thoroughly and comprehensively, we further analyzed its complete genome (Figure 1A) and submitted it. The total number of base pairs contained in the complete genome of SDTB038 was 3,929,791, and the GC content was 47.26%. The whole gene sequences of strain SDTB038 were submitted to the National Center for Biotechnology Information (NCBI) database with the accession numbers SUB6310808. The number of genes annotated by specific genes in each database was as follows NR (3944), GO (2707), COG (2900), KEGG (3833), Swiss-Prot (3314), pfam (2707), TCDB (450), and CAZy (159).

Gene functional annotation results from the main databases. Gene Ontology (GO) is a widely used and important tool in the field of bioinformatics. It is an international standardized classification system for gene function descriptions. It describes our understanding of the biological field from three aspects (Figure 1B). The biological process (BP) genes are further subdivided into 24 subfunctions, with genes involving metabolic and cellular processes numbering at 1602 and 1,559, respectively. Genes in cell components (CCs) were subdivided into 10 subfunctions, with the largest number of genes in cells and cell parts, both with 1,036; the genes in molecular functions (MFs) were also subdivided in 10 subfunctions, and the number of genes involved in catalytic activity reached 1,550. Based on GO annotation, the most abundant genes of strain SDTB038 in BP, CC and MF were metabolism, catalysis and cell function.

Kyoto Encyclopedia of Genes and Genomes (KEGG) is a database for genome deciphering with which the possible metabolic processes or functions of SDTB038 were obtained by comparative analysis. ABC transporters (127), two-component systems (114) and other pathways had a high correlation with the genome of strain SDTB038. In addition, by comparison with known functional sequences, these pathways were divided into six categories, as shown in Figure 1C. SDTB038 had the highest abundance of genes involved in metabolism.

Cluster of Orthologous Groups of proteins (COG) is constructed according to the phylogenetic relationships of coding proteins in the complete genome. The biological function of SDTB038 was also revealed by COG annotation. A total of 3,337 genes were annotated to 25 COG families (Figure 1D), which were related to metabolism, cell process and signaling, genetic information storage and processing. The analysis showed that SDTB038 had a strong metabolic function, and the annotated genes related to metabolic function accounted for 42.97%. The large proportion of genes involved in cell processes and signaling was mainly related to cell membrane, wall, and capsule biosynthesis and signal transduction mechanisms, accounting for more than 5.5%. The number of genes related to genetic information storage and processing was second only to those in metabolic function, accounting for 22.95% of the total annotated genes, of which transcription-related genes accounted for 8.6%. In addition, the conventional function accounted for 9.14%, and the unknown function accounted for 6.23%, indicating that the potential abilities of the biocontrol bacterium SDTB038 remains to be further developed.

Gene clusters for secondary metabolite synthesis. Gene clusters associated with secondary metabolite synthesis were annotated using the antiSMASH6.0.1 database. The results showed that SDTB038 contained biosynthesis gene clusters including those for macrolactin H, bacillaene, fengycin, difficidin, *bacillus* bactin, bacilysin and surfactin. Although that surfactin showed 86% similarity to the reported secondary metabolite synthesis gene cluster, the similarity of other gene clusters was 100% (Table 4). The results showed that the SDTB038 genome has genes that can be used for the biosynthesis, regulation and transport of antimicrobial substances, and there were many genes beneficial to plant growth, that were related to antimicrobial and growth-promoting potential.

**Table 4:** Biosynthetic gene clusters of secondary metabolite in strain of SDTB038 genome.

Gene cluster type	Metabolite	Gene cluster ID similarity (%)	Gene location Start – end	Size(bp)
nrps	surfactin	BGC0000433(86%)	323,410–387,387	63,978
transatpks	macrolactin H	BGC0000181(100%)	1,384,086–1,471,921	87,836
transatpks-nrps	bacillaene	BGC0001089(100%)	1,691,450–1,792,015	100,566
transatpks-nrps	fengycin	BGC0001095(100%)	1,865,757–2,000,046	134,290
transatpks	difficidin	BGC0000176(100%)	2,282,382–2,376,174	93,793
bacteriocin-nrps	bacillibactin	BGC0000309(100%)	3,000,878–3,052,669	51,792
other	bacilysin	BGC0001184(100%)	3,588,979–3,630,397	41,419

### Isolation and identification of pathogens

The results showed that FOL failed to amplify the target band, and the isolated strain (FO-1) amplified the target band at the position of nearly 1,000 bp, which was consistent with the results of Hirano and Arie. After Blast comparison, the *ITS* gene of the isolated strain was 100% similar to that of *F. oxysporum* AM4. In the *ITS* gene system tree (Supplementary Figure S1), the isolates (FO-1) and *F. oxysporum* AM4 were clustered in the same branch.

After root inoculation, the stem base exhibited light brown stripe rot and brown depressions, and the plants showed lodging. The pathogenic fungi were isolated from the diseased plants. The isolated and purified strains were similar to the original strain in morphology and biological identification results. According to the growth morphology and molecular biology identification results, the isolated strain was identified as F. *oxysporum* f. sp. *radicis-lycopersici* (FORL).

### Antifungal effect of SDTB038

The results showed that the inhibition zone width of SDTB038 fermentation broth with different concentrations was 5.68–7.66 mm, which indicated that SDTB038 had activity against FORL. The statistical analysis of the antifungal band width showed that the effect of SDTB038 at 10^7^ and 10^8^ CFU/ml concentrations was significantly higher than that of other concentrations (Table 5). The maximum inhibition zone width was 7.66 ± 0.23 mm at 10^8^ CFU/ml (Supplementary Figure S2).

**Table 5:** Antibacterial activity of SDTB038 gradient fermentation broth.

Fermentation broth concentration (CFU/mL)	Bacteriostatic diameter (mm)
_10_10	5.78 ± 0.21c
10^9^	6.72 ± 0. 11b
10^8^	7.66 ± 0. 23a
10^7^	7.43 ± 0. 09a
10^6^	5.68 ± 0.24c
10^5^	6.25 ± 0.37bc

Values are means ± SD of 3 experiments. The lowercases represent significant level (*p* = 0.05) according to the Duncan’s test.

SDTB038 at different concentrations had good control effects on Fusarium crown and root rot of tomato pathogens, and SDTB038 at 10^7^ and 10^8^ CFU/ml had the best control effect.

### Determination of the growth temperature of SDTB038

**Figure 2:**
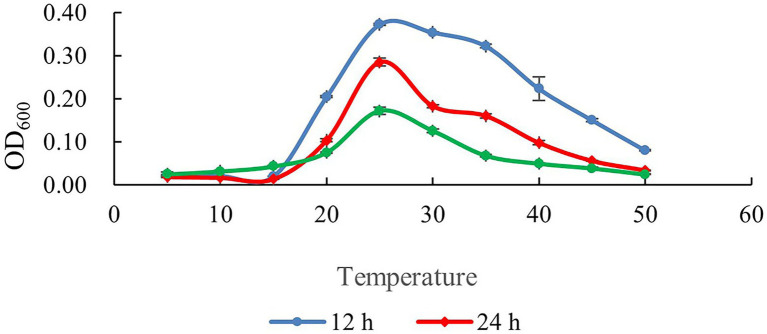
Determination of growth temperature of SDTB038. Blue: growth curve of SDTB038 cultured for 12 h; Red: growth curve of SDTB038 cultured for 24 h; Green: growth curve of SDTB038 cultured for 48 h.

The growth curves of SDTB038 cultured at different temperatures for 12, 24 and 48 h are shown in Figure 2. Under 20°C, the activity of SDTB038 cultured for 12, 24, 48 h was low and the growth rate was slow. Between 20°C and 30°C, the growth rate of SDTB038 cultured in three periods was the fastest, and the growth rate reached the peak around 25°C. When the temperature was higher than 30°C, the growth rate of SDTB038 cultured in three periods decreased significantly. The growth rate is slow to flat after 40°C. The tests indicated that the optimal growth temperature range of SDTB038 was 25–30°C. When the temperature was lower than 20°C or higher than 35°C, the growth rate of SDTB038 decreased significantly. Although the growth slowed down at low temperature, SDTB038 could still grow at 5°C, it indicated that SDTB038 could colonize in tomato roots at low temperature.

### Indole acetic acid production capacity of SDTB038

SDTB038 was cultured in 20 ml liquid LB containing 1% L-tryptophan for 2 days. Two milliliters of supernatant were taken, was added to 4 ml Salkowski reagent and two drops of orthophosphoric acid. After bathing in 28°C water bath for 2.5 h, it was found that the mixture showed light pink, while the control group without biocontrol bacteria showed light yellow. Meanwhile, transparent halos were not observed on NBRIP plates. Therefore, the experiment showed that SDTB038 could produce indoleacetic acid, promote seed germination and plant growth, prevent and treat diseases, and have plant health effects. However, SDTB038 had no phosphorus-solubilizing effect.

### Hydroponic culture experiment with tomato seedlings

**Figure 3:**
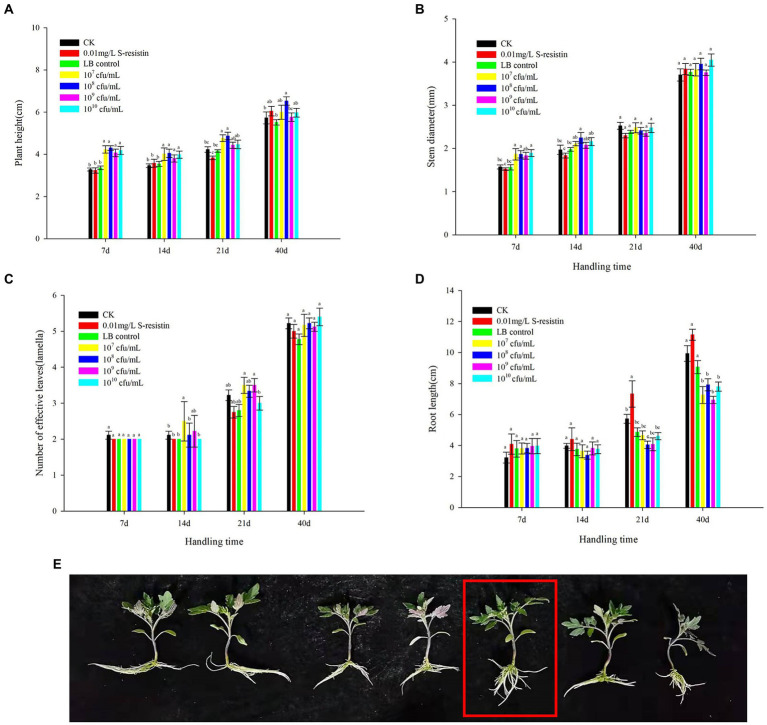
Effects of SDTB038 on tomato seedlings. **(A)** Plant height; **(B)** Stem diameter; **(C)** Number of effective leaves; **(D)** Root length; **(E)** Effects of fermentation broth on root length of tomato plants. The different lowercases represent significant level (*p* = 0.05) according to the Duncan’s test.

In the greenhouse hydroponic experiment, it can be seen from Figure 3A that 10^7^–10^10^ CFU/ml SDTB038 resulted no significant difference in plant height at the statistical level at 7 and 14 days after treatments. Among them, 10^8^ CFU/ml had the best effect after processing for approximately 7 days, and the plant height of tomato was 4.05 cm; 10^7^ CFU/ml had the best effect after processing for approximately 14 days, and the plant height of tomato was 4.39 cm. At 21 d after treatment, the 10^7^ and 10^8^ CFU/ml SDTB038 treatments resulted in plant height that were significantly higher than those under the water, LB and S-inducible control treatments, and the plant height of the 10^8^ CFU/ml SDTB038 treatment group was 4.87 cm. After 40 days of treatment, the height under the 10^8^ CFU/ml SDTB038 treatment was obviously higher than that of the water and LB control treatments, at 6.53 cm. Except for the 10^8^ CFU/ml treatment, there was no obvious change among the other treatments.

The effect of each treatment on the stem diameter of tomato seedlings was mainly reflected in the early stage of treatment (7 and 14 d after treatment). After approximately 7 days, the 10^7^, 10^8^ and 10^10^ CFU/ml SDTB038 treatments resulted in a significantly larger diameter than those under the clean water, LB and S-selectin control treatments; on the 14th day after treatment, the diameter under the 10^8^ CFU/ml SDTB038 treatment was obviously different from that under water, LB and the S-induced control. There was no obvious change between treatments at 21 and 40 days after treatment (Figure 3B).

The fermentation broth treatment had little effect on the number of effective leaves. In the statistical analysis results, there was no difference in the effect between the two groups in the first 7 days; After processing for approximately 14 days, the number of effective leaves in the 10^7^ CFU/ml SDTB038 experimental group was obviously higher than that in the water, liquid LB and S-inducible control treatments. However, 21 d after treatment, there was no obvious change between the treatment with 10^7^–10^9^ CFU/ml SDTB038 fermentation broth and the control treatment with clear water, liquid LB and S-selectin. There was no obvious change among treatments 40 days after treatment (Figure 3C).

The effect of each treatment on root length was mainly reflected in the later treatment stage (21 and 40 d). There was no obvious change in the statistical level between the seven groups at 7 and 14 d after treatment. Twenty-one days after treatment, the promoting effect of S-selectin treatment on root length was significantly higher than that of the other treatments. After processing for approximately 40 days, water, liquid LB treatment and inducer treatment effects were better. However, when the fresh weight and dry weight of seedling roots were measured, it was found that although the seedling roots in the water treatment, liquid LB treatment and S-selectin treatment were longer than those in the fermentation broth treatment at 40 d after treatment, the weighing measurement results (Table 6) showed that the fresh weight of roots under the 10^8^ CFU/ml SDTB038 fermentation broth treatment was 1.13 g, which was 59.15%, 73.85% and 24.18% higher those under the water treatment, liquid LB treatment and S-selectin treatment, which were 0.71 g, 0.65 g and 0.91 g, respectively (Figures 3D,E). Statistical analysis showed that 10^8^ CFU/ml SDTB038 fermentation broth had the same effect on roots as the s-selectin treatment, but resulted in significantly higher weight than that under the water and liquid LB treatments. After drying the seedling roots to constant weight, the root dry weight under the 10^8^ CFU/ml SDTB038 fermentation broth treatment was 0.065 g, which was 51.16%, 62.50% and 47.73% higher than the 0.043 g, 0.040 g and 0.044 g of the water, liquid LB and S-inducible treatments, respectively. At the statistical level, the 10^8^ CFU/ml SDTB038 fermentation broth treatment resulted in significantly higher weight than the water, liquid LB and S-inducible treatments.

**Table 6:** Effects of SDTB038 on fresh and dry weight of tomato seedlings.

Treatment	Fresh weight		Dry weight	
	Root(g)	Aerial parts (g)	Root (g)	Aerial parts (g)
1	0.71 ± 0.06bc	1.38 ± 0.12b	0.043 ± 0.004b	0.12 ± 0.014bc
2	0.91 ± 0.06abc	1.47 ± 0.08ab	0.044 ± 0.003b	0.12 ± 0.006bc
3	0.65 ± 0.04c	1.18 ± 0.09b	0.040 ± 0.002b	0.10 ± 0.009c
4	1.02 ± 0.11ab	1.69 ± 0.11ab	0.058 ± 0.006ab	0.15 ± 0.008ab
5	1.13 ± 0.13a	1.91 ± 0.12a	0.065 ± 0.006a	0.17 ± 0.009a
6	0.86 ± 0.13abc	1.61 ± 0.22ab	0.051 ± 0.007ab	0.15 ± 0.019ab
7	1.05 ± 0.21ab	1.99 ± 0.35a	0.060 ± 0.012ab	0.17 ± 0.028a

1. Water control; 2. 0.1 mg/l S-dactin; 3. LB control; 4. 10^7^ CFU/ml fermentation broth; 5. 10^8^ CFU/ml fermentation broth; 6. 10^9^ CFU/ml fermentation broth; 7. 10^10^ CFU/ml fermentation broth. Values are means ± SD of three experiments. The different lowercases represent significant level (*p* = 0.05) according to the Duncan’s test.

### SDTB038 can induce cryogenic genes and reduce active reactive oxygen species accumulation in tomato

**Figure 4:**
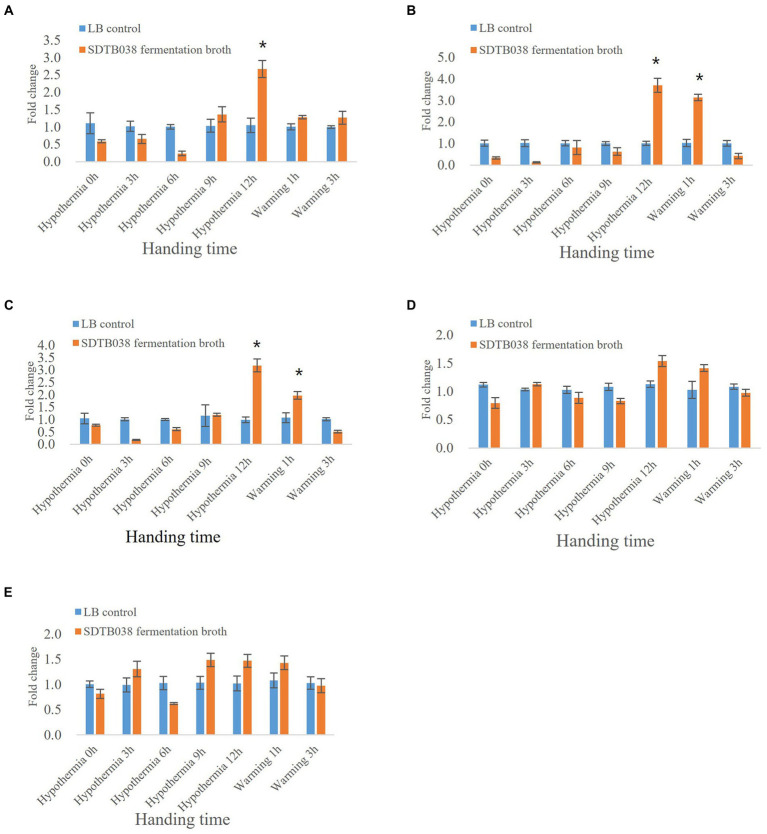
Expression of SDTB038 on antifreeze genes at low temperature. **(A)** *SIMPK3* gene expression; **(B)** *SIHSP17.7* gene expression; **(C)** *SLMYB7* gene expression; **(D)** *SICPK8* gene expression; **(E)** *SIHY5* gene expression. Asterisks indicate significant differences between tomato plants inoculated with SDTB038 broth and those inoculated with liquid LB. The different lowercases represent significant level (*p* = 0.05) according to the Duncan’s test.

Through low-temperature stress treatment of tomato, we studied the expression of antifreeze genes in strain SDTB038. The *SIMPK3* gene regulates ROS homeostasis by activating the cell antioxidant system and regulating the transcription of stress-related genes, thus acting as a positive regulator in the low-temperature stress response. The expression of *SIMPK3* in tomato tissues at 0, 3, 6 and 9 h after applying SDTB038 fermentation broth was not different from that in the control group. The expression of *SIMPK3* in tomato tissues was upregulated 12 h after low temperature treatment. After rewarming, *SIMPK3* gene expression gradually decreased (Figure 4A). The *SIHSP17.7* gene and *SLMYB7* gene could improve the regulation of cells under low temperature in tomato and regulate the ROS content in plants. After 12 h of low-temperature treatment, the expression levels of the *SIHSP17.7* gene and *SLMYB7* gene were upregulated in tomato plants treated with SDTB038 fermentation broth. The expression of both genes increased gradually at 1 h after rewarming. After 3 h of rewarming, the expression levels of the *SIHSP17.7* gene and *SLMYB7* gene gradually decreased and returned to normal levels (Figures 4B,C). There was no obvious change in the expression of the *SICPK8* gene and *SIHY5* gene between tomato tissues at different time points (Figures 4D,E).

**Figure 5:**
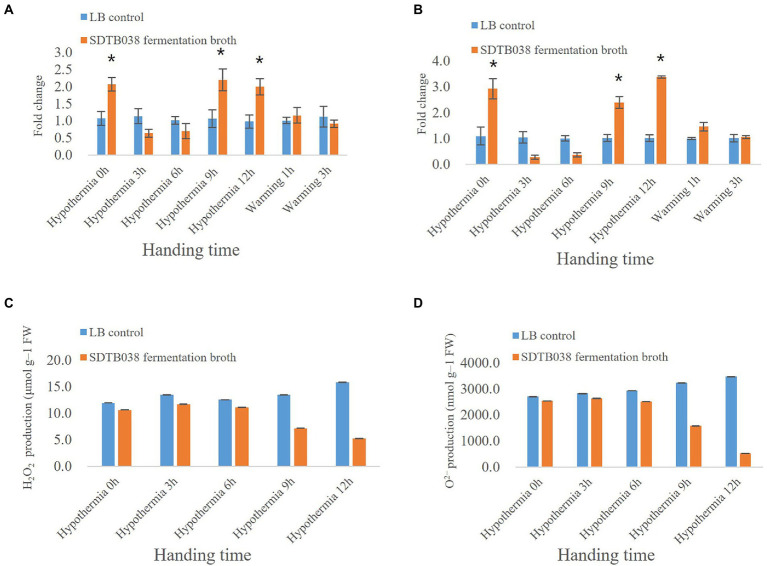
Expression of enzyme activity genes and changes of H_2_O_2_ and O^2−^ Content. **(A)** *CAT* expression; **(B)** *SOD* expression; **(C)** Changes of H_2_O_2_ Content; **(D)** Changes of O^2−^ Content. Asterisks indicate significant differences between tomato plants inoculated with SDTB038 broth and those inoculated with liquid LB. The different lowercases represent significant level (*p* = 0.05) according to the Duncan’s test.

By measuring the activity of superoxide dismutase (SOD) and catalase (CAT), it was found that both were increased at 0 h, which may have been due to the application of SDTB038 fermentation broth to promote the production of SOD and CAT enzymes. At 3 and 6 h, *SOD* and *CAT* were affected by low-temperature stress, downregulating their expression. At low temperature for 9 and 12 h, both of the expression levels were upregulated, with a significant difference from those under the liquid LB treatment, because the application of SDTB038 fermentation broth promoted the expression of antifreeze genes (Figures 5A,B).

**Figure 6:**
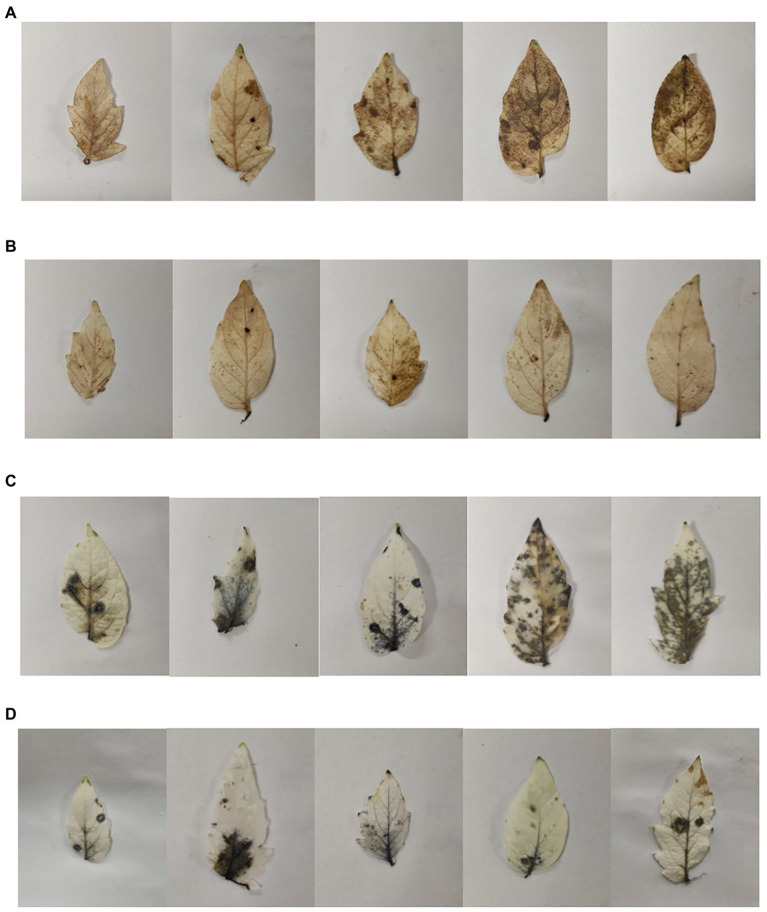
Comparison of ROS accumulation in leaves. **(A)** LB control H_2_O_2_ staining; **(B)** SDTB038 fermentation broth H_2_O_2_ staining; **(C)** LB control O^2−^ staining; **(D)** SDTB038 fermentation broth O^2−^ staining.

At the same time, we evaluated the accumulation of ROS in leaves at low temperature. The contents of H_2_O_2_ and O^2−^ were detected by kits. The contents of H_2_O_2_ and O^2−^ and in tomato plants treated with liquid LB gradually increased and ROS accumulated under low temperature conditions. However, the contents of H_2_O_2_ and O^2−^ in tomato plants treated with SDTB038 fermentation broth increased first and then decreased, and the contents of H_2_O_2_ and O^2−^ were the lowest at the low temperature treatment for 12 h (Figures 5C,D). At the same time, H_2_O_2_ and O^2−^ were detected by staining, and the staining increased first and then decreased, which was consistent with the content determination (Figure 6). It can be concluded that strain SDTB038 could promote the expression of antifreeze genes and reduce the accumulation of ROS under low temperature conditions.

### Colonization test of strain SDTB038

Natural resistance of the biocontrol strain SDTB038. In the natural resistance test of biocontrol bacteria, the control plate without antibiotics grew single colonies on the surface of the medium after 12 h of culture, and no single colonies grew on the plate with different concentrations of rifampicin, ampicillin and kanamycin. Therefore, SDTB038 had no natural resistance to antibiotics such as rifampicin.

Rifampicin resistance in soil microbial populations. In the resistance test of the soil microbial population to rifampicin, on the control plate without rifampicin, the soil suspension before and after dilution showed the growth of soil microorganisms. In terms of surface morphology, mainly bacterial microorganisms were present, while there was no trace of microbial growth on the rifampicin plate with 100 mg/l.

Morphological observation, physiological and biochemical characterization and molecular biological identification of the rifampicin-labeled strain. After the genetic stability test of the resistant strains that could grow normally on 200 mg/l rifampicin plates, the colony morphology was observed by streaking. Under the same culture time and conditions, compared with the wild-type strain, the growth of the rifampicin-labeled strain was slower, but that of the single colony was the same as that of the wild-type strain, which was white on the medium plate, with smooth and neat edges, and wrinkled bulges in the middle.

The physiological and biochemical results showed that the rifampin-labeled strain was gram-positive bacterium that could hydrolyze gelatin and starch, did not produce pyocyanin, hydrogen sulfide or fluorescent pigments, and could not use citric acid or malonic acid. The rifampin-labeled strain was positive in the V-P test, contact enzyme test, nitrate reduction test and anaerobic growth test, and negative in the methyl red test, which was the same as the results of the previous determination of wild strains in the laboratory. DNAMAN software was used to compare the 16S rRNA gene and *gyrB* gene fragments of rifampicin-resistant marker strains and wild strains, and the results showed 100% identity.

Colonization characteristics of SDTB038. The dynamic colonization results of SDTB038 in different parts of tomato rhizosphere soil and tomato plants are shown in Supplementary Figure S3. The bacterial amount decreased with time in the early stage and gradually stabilized in the later stage.

SDTB038 had the highest amount of recovered bacteria in rhizosphere soil on the first day after root irrigation, which was 2.05 × 10^9^ CFU/g. On the 4th day, it decreased to the first low of 3.42 × 10^7^ CFU/g and increased on the 5th day. Subsequently, with increasing culture time, colony colonization decreased gradually. At the later stage of the experiment (28, 40, and 60 d), the colonization rate of biocontrol bacteria in tomato rhizosphere soil tended to be stable at 2.89 × 10^6^ CFU/g and 5.91 × 10^6^ CFU/g. However, the colonization rate of SDTB038 in tomato roots was 2.24 × 10^8^ CFU/g on the first day after root irrigation, and rapidly decreased to the first low value on the third day. The colonization amount was 1.92 × 10^7^ CFU/g, and then slowly increased. The colonization amount increased to 1.23 × 10^8^ CFU/g on the sixth day, but then sharply decreased to a new low value of 2.00 × 10^7^ CFU/g on the seventh day. On the 14th day, the colonization amount of biocontrol bacteria peaked 3.89 × 10^9^ CFU/g and then decreased over time and gradually stabilized on the days 40 and 60.

The colonization of SDTB038 in the stem bases of tomato was detected on the 2nd day after root irrigation, and the amount of bacteria was 5.00 × 10^6^ CFU/g. There was a low value on the 4th day, and the amount of bacteria was 3.67 × 10^6^ CFU/g. The colonization continued to increase over the next 2 days, a new low value appeared on the 7th day, and the amount of bacteria was 1.52 × 10^6^ CFU/g. The colonization of biocontrol bacteria peaked 14th day, and the amount of bacteria was 4.07 × 10^9^ CFU/g. After 21 days, the amount of bacteria decreased sharply, and then the colonization became stable. The colonization of SDTB038 in tomato leaves was divided into two parts, original leaves and new leaves, after 14 days of colonization. It can be seen from the figure that the colonization of biocontrol bacteria in the original true leaves and new leaves was similar to that in roots and stems, and peaked at 14 d after treatment. The colonization amount in the original true leaves was 9.13 × 10^7^ CFU/g, and that in the new leaves was 2.07 × 10^9^ CFU/g. The change trends of the two were consistent. The colonization amount decreased sharply at 21 d after treatment, and then stabilized after 21 d. On the 60th day, the original true leaf colonization rate was 6.56 × 10^4^ CFU/g, and the new leaf colonization rate was 4.51 × 10^4^ CFU/g.

Scanning electron microscopy observations. Supplementary Figure S4 shows our SEM (5,000 × SEM) observations. Supplementary Figures S4A,B shows the electron microscopy images and figures of tomato roots after 7 d of fermentation broth treatment and LB control treatment, respectively. Supplementary Figures S4C,D shows the electron microscopy images and figures of tomato the stem base after 7 d of fermentation broth treatment and LB control treatment, respectively. Supplementary Figures S4E,F shows the electron microscopy images and figures of tomato roots after 60 d of fermentation broth treatment and LB control treatment, respectively. Supplementary Figures S4G,H shows the electron microscopy images and figures of tomato the stem base after 60 d of fermentation broth treatment and LB control treatment, respectively. As shown in Supplementary Figure S4, compared with the control treatment, a large amount of *Bacillus* adhesion was observed in the root and stem base of tomato in the fermentation broth treatment group.

### Field efficacy of SDTB038 against Fusarium crown and root rot of tomato

In the field efficacy tests, the disease indexes of the water treatment, LB treatment and microbial fertilizer treatment were 33.61, 32.78 and 27.37%, respectively. The disease index of the SDTB038 with 10^8^ CFU/ml was 19.16%, which was 42.99, 41.55 and 30% lower than that of water treatment, LB control and microbial fertilizer treatment, respectively. The disease index of difenoconazole treatment was 12.22%, which was 63.64, 62.72 and 55.3% lower than that of clean water, LB control and microbial fertilizer treatment, respectively. The control effect of the difenoconazole treatment was 63.63%, significantly higher than 10^8^ CFU/ml SDTB038 bacterial liquid and microbial fertilizer treatment. The control effect of SDTB038 at 10^8^ CFU/ml was 42.98%, which was significantly higher than that of microbial fertilizer treatments (control effect was 19.33). The above results showed that SDTB038 could better control Fusarium crown and root rot of tomato compared with microbial fertilizer containing *B. subtilis* (Table 7).

**Table 7:** The disease index and control effect of Fusarium crown and root rot of tomato.

Treatment	Index(%)	Control effect(%)
1	33.61 ± 1. 82	
2	32.78 ± 2.78	
3	27.37 ± 1.32	19.33 ± 4.33a
4	12.22 ± 1.82	63.63 ± 5.42b
5	19.16 ± 2.20	42.98 ± 6.56c

1. Water control; 2. LB control; 3. microbial fertilizer; 4. 75 g ha^−1^ Difenoconazole (10% water dispersible granules); 5. 10^8^ CFU/ml fermentation broth. The different lowercases represent significant level (*p* = 0.05) according to the Duncan’s test.

## Discussion

**Figure 7:**
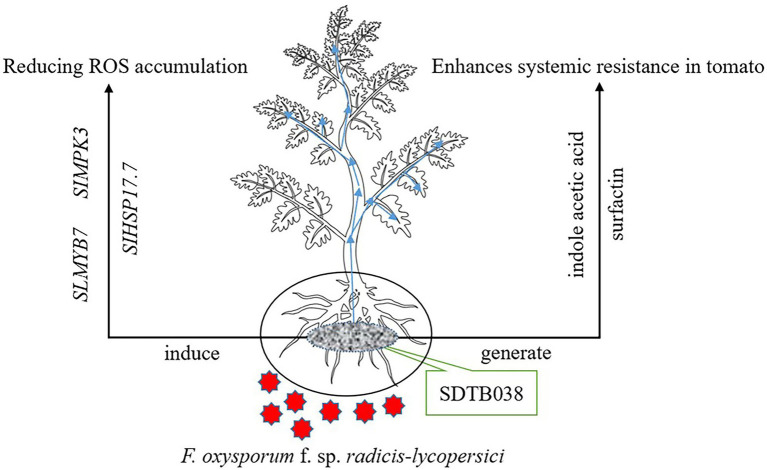
SDTB038 action mechanism diagram. Strain SDTB038 colonizes in tomato roots and can be moved to the rhizomes and leaves, which can induce the expression of antifreeze genes, produce indoleacetic acid and surfactin, and prevent and treat diseases.

Genome-wide sequencing analysis is of great significance to understand the mechanisms of microbial control and disease prevention. This method can locate functional genes and predict the gene clusters for secondary metabolite synthesis, which is conducive to the rapid identification of organisms with biocontrol potential and the development of engineered strains (Chun et al., 2019). These methods help reveal biocontrol mechanisms at the genome level, and lays the foundation for the in-depth study and utilization of biocontrol bacteria (Chen et al., 2007). Wang et al. (2019) proved that WRN014 and other *B. velezensis* strains had potential as growth-promoting bacteria and biopesticides through whole-gene sequencing. A *B. velezensis* YYC strain was isolated from the tomato rhizosphere by Yan Y. et al. (2021), who found that the genome size was 3,973,236 bp and consisted of 4,034 genes in total, with a mean GC content of 46.52%. In this study, the whole genome of SDTB038 was sequenced by combining second-generation and third-generation sequencing technology. The genome size was 3,929,791 bp, and its GC content was 47.26%. In addition, through software prediction, seven biosynthetic gene clusters of secondary metabolites were found, proving that *B. velezensis* can reduce or even eliminate the accumulation of ROS under low temperature stress. The strain could produce indole acetic acid and surfactin, which play a role in the prevention and treatment of diseases (Figure 7).

Fusarium crown and root rot of tomato is a soilborne disease that has spread all over the world and seriously threatens tomato quality and production (Chu et al., 2021). After decades of research abroad, a variety of resources have been identified, including microorganisms such as *B. amyloliquefaciens*, *B. cereus*, and *Rhizobium*. Debbi et al. (2018) verified that *Trichoderma harzianum* had different degrees of inhibition of FORL, indicating its potential as part of a biocontrol strategy against the disease. Moreover, *B. velezensis* is considered an important PGPR, could product many bioactive primary metabolic products, and inhibiting plant diseases and harmful substances (Rabbee et al., 2019). Rabbee et al. (2019) showed that *B. velezensis* played an important role in inhibiting pathogens and promoting plant growth. Our research was similar to that of Liu et al. (2020), who found that *B. velezensis* produced antimicrobial substance that showed antimicrobial activity against pathogenic fungi and bacteria. Results indicated that different concentrations of SDTB038 fermentation broth inhibited the mycelial growth of Fusarium crown and root rot of tomato.

PGPR are beneficial bacteria that can colonize and promote plant growth, and inhibit or reduce the threat of plant diseases (Sarwar et al., 2020). Tests have indicated that plant rhizosphere bacteria can promote the dissolution of phosphorus and potassium, inhibit pathogen, improve stress resistance, and produce IAA and other plant hormones, thereby increasing crop yields (Agha et al., 2022). The study of PGPR, mainly including *Mesorhizobium* and *Allorhizobium,* intracellular PGPR, and *Bacillus*, *Pseudomonas* and *Azotobacter* extracellular PGPR (Li et al., 2020), has attracted the attention of scholars at home and abroad. To screen salt-tolerant *Bacillus* strains and growth-promoting microorganisms, Medeiros and Bettiol (2021) evaluated: the production of iron carriers and indoleacetic acid (IAA) and solubilization of phosphate. A study found that SDTB038 could produce indole acetic acid. However, SDTB038 had no phosphorus-solubilizing effect. Through greenhouse hydroponic experiments, the results showed that SDTB038 had plant growth promoting effects, we speculate that indole acetic acid may play a role in regulating plant growth.

Low temperature is one of the adverse factors affecting plant growth, resulting in loss of plant (mainly crop) economy and yield. Overall, the increase and decrease in ROS in plant cells is a dynamic equilibrium process. However, when plants receive low temperature stress, this balance will be broken, resulting in a large accumulation of ROS in plants, thereby causing damage to cells (Zhang et al., 2019). Therefore, it is very important to control the production of ROS in plants and eliminate the accumulation of ROS under low temperature (Wang et al., 2020). Heidari et al. (2021) showed that oxidative stress can be induced under low temperature, and EBR treatment may increase antioxidant enzymes and improve tomato growth by reducing the content of reactive oxygen species. Wang et al. (2020) found that endogenous melatonin could reduce the content of ROS and increase antioxidant enzymes in tomato leaves under low-temperature stress, while exogenous melatonin could not reduce the content of ROS. Han et al. (2020) showed that *SlHY5* improved the tolerance of plants to low temperature and can be used to promote the improvement of cold resistance in tomato. However, in this experiment, the tomato plants treated with SDTB038 fermentation broth failed to induce the up-regulation of *SlHY5* gene, which could not induce low temperature tolerance in these tomatoes. At the same time, our results are similar to those of Wang et al. (2017), who found that the *SIMPK3* gene can increase tomato tolerance to low temperature, and regulate plant hormone concentrations and antioxidant enzyme activity to enhance the cold tolerance of tomato plants. Experimental studies have shown that SDTB038 can induce the expression of *SIHSP17.7* and *SLMYB7* genes, and regulate ROS contents in plants by regulating nonenzymatic antioxidant systems such as those involving SOD and CAT.

The colonization process of biocontrol bacteria involves biofilm formation, flagella, fimbriae, motility, chemotaxis and so on. It is closely related to light, temperature and soil microorganisms (Abdallah et al., 2020). Huang et al. (2022) systematically explored the potential adhesion substances of PGPR strains, and the adhesion process required for root colonization was clarified. This helps strengthen rhizosphere stability. In this experiment, the colonization dynamics of rifampicin-resistant marker strains in rhizosphere soil and tomato roots, stems and leaves were also studied. The results indicated that SDTB038 could colonize normally in soil and plants and grow steadily. Yan H. et al. (2021) showed that *B. velezensis* had good antibacterial activity, which could effectively control potato late blight in greenhouse and field, and promote the growth of potato plants, the field control effect was 42.43%. Zhou et al. (2021) found that the combination of *Trichoderma virens* (Tvien6) and *B. velezensis* (X5) could control tomato bacterial wilt. *B. velezensis* (X5) could promote tomato plant growth and enhance defense enzyme activity. In this study, it was found through field experiments that SDTB038 had good control effects on Fusarium crown and root rot of tomato. The control effect of 10^8^ CFU/ml SDTB038 fermentation broth was the best, which was 42.98%.

## Conclusion

In the present study, we investigated the ability of *B. velezensis* SDTB038 to promote tomato plant growth and prevent Fusarium crown and root rot of tomato. *B. velezensis* had antifungal activity against the Fusarium crown and root rot pathogen of tomato according to whole genome prediction. It could produce indole acetic acid, had a stable colonization state, induce the expression of antifreeze genes in tomato plants under low temperature and reduce the accumulation of ROS. Meanwhile, it was found through field experiments that SDTB038 had good control effects on Fusarium crown and root rot of tomato. The control effect of 10^8^ CFU/ml SDTB038 fermentation broth was the best, which was 42.98%.

## Data availability statement

The data provided in the study are stored in the National Center for Biotechnology Information (NCBI) database, with registration number SUB6310808.

## Author contributions

QC and YQ performed the experiments, analyzed the data, and wrote the manuscript. YY collected pathogen isolates. KW revised the manuscript. HW designed the experiments, supervised the project, and wrote the manuscript. All authors contributed to the article and approved the submitted version.

## Funding

This research was supported by the National Natural Science Foundation of China (32102259) and the Central Guidance of Local Science and Technology Development Special Foundation of Shan-dong Province (YDZX2021088).

## Conflict of interest

The authors declare that the research was conducted in the absence of any commercial or financial relationships that could be construed as a potential conflict of interest.

## Publisher’s note

All claims expressed in this article are solely those of the authors and do not necessarily represent those of their affiliated organizations, or those of the publisher, the editors and the reviewers. Any product that may be evaluated in this article, or claim that may be made by its manufacturer, is not guaranteed or endorsed by the publisher.

## Footnotes

^1^ http://topaz.gatech.edu/
