# Supplementary material for: Biocontrol activity and action mechanism of Bacillus velezensis strain SDTB038 against Fusarium crown and root rot of tomato
Source: Front Microbiol. 2022 Sep 2;13:994716. doi: 10.3389/fmicb.2022.994716 (PMC9479544; doi:10.3389/fmicb.2022.994716)
Supplement: Supplementary file 1 [file Table_1.DOCX]

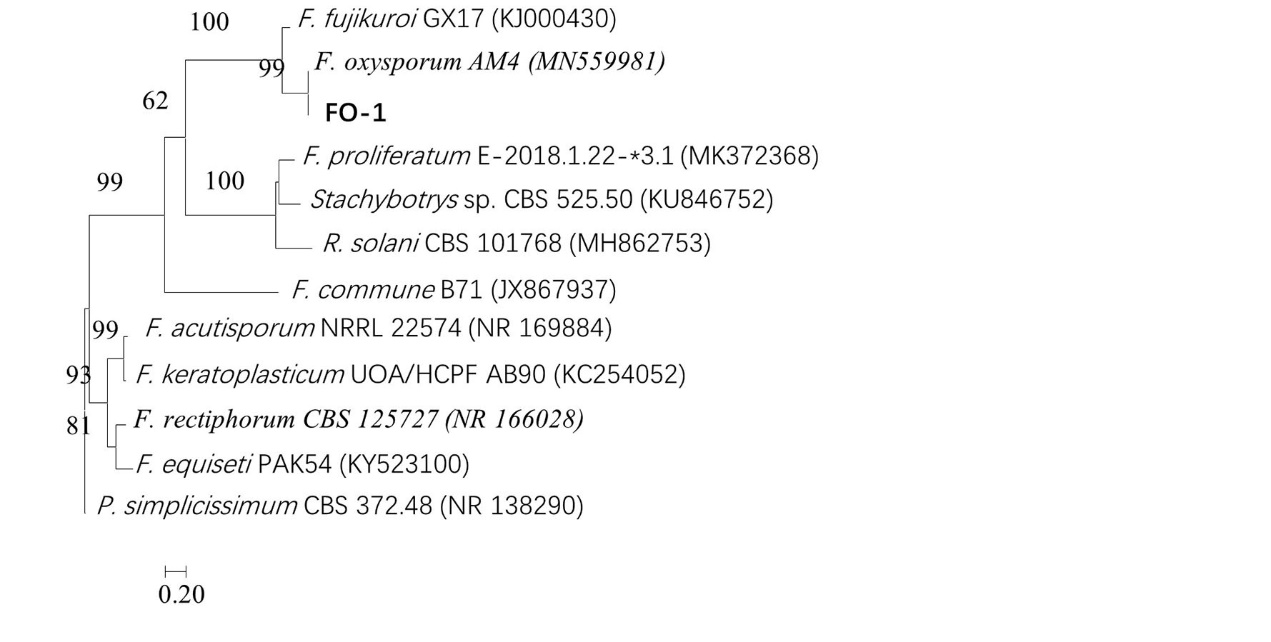


Supplementary Figure 1 Phylogenetic tree based on *ITS* gene

Phylogenetic tree based on *ITS* sequences showed the relationship between isolated strain (FO-1) and *Fusarium*.


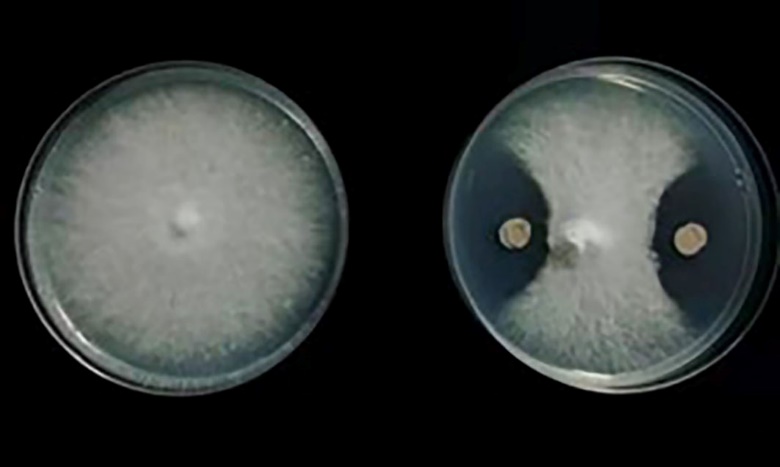


Supplementary Figure 2 Strain SDTB038 and FORL were stand-off cultured

The left side was blank control; effect of SDTB038 on FORL at 10^8^ CFU/mL on the right side.


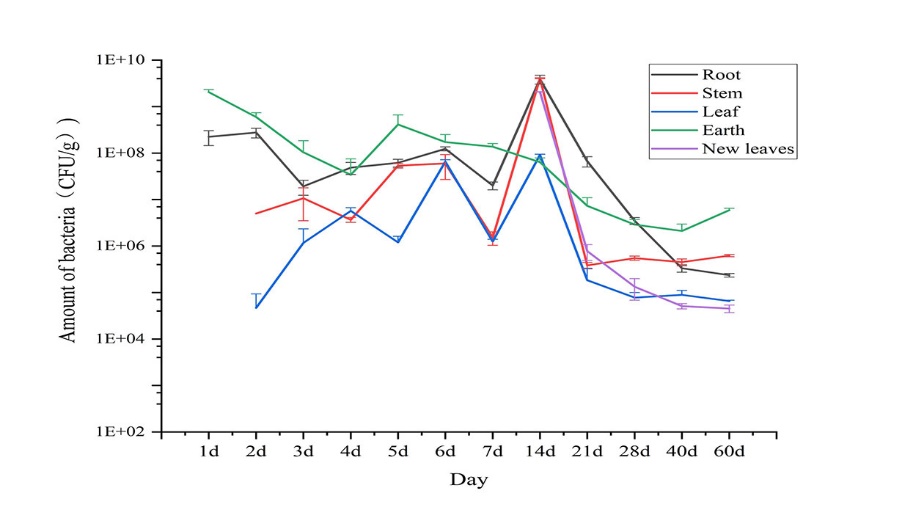


Supplementary Figure 3 Colonization dynamics of SDTB038

Black: root；Orange：stem; Blue: leaf; Green: earth; Purple: new leaves.


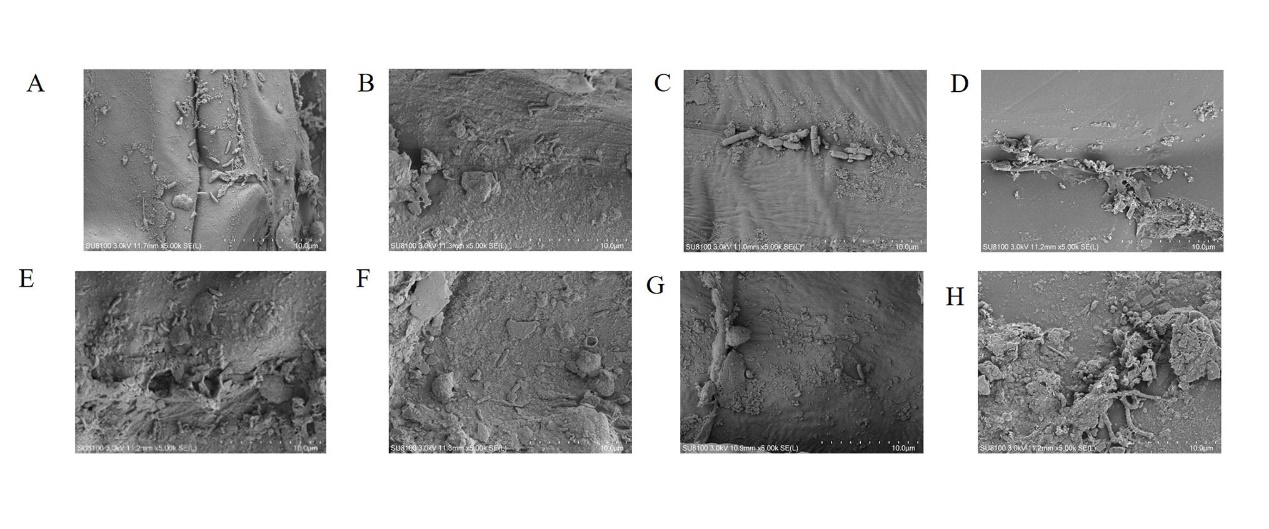


Supplementary Figure 4 SEM of SDTB038 attached to plant roots and stems

A: Root colonization on the 7th day (treatment of SDTB038); B: Root colonization on the 7th day (CK); C: Stem colonization on the 7th day (treatment of SDTB038); D: Stem colonization on the 7th day (CK); E: Root colonization on the 60th day (treatment of SDTB038); F: Root colonization on the 60th day (CK); G: Stem colonization on the 60th day (treatment of SDTB038); H: Stem colonization on the 60th day (CK).
